# CD271+ Mesenchymal Stem Cells as a Possible Infectious Niche for *Leishmania infantum*

**DOI:** 10.1371/journal.pone.0162927

**Published:** 2016-09-13

**Authors:** Carolina S. Lopes, Nada Daifalla, Bikul Das, Valdo Dias da Silva, Antonio Campos-Neto

**Affiliations:** 1 The Forsyth Institute, Cambridge Massachusetts, United States of America; 2 Department of Biochemistry, Pharmacology, Physiology and Molecular Biology, Institute for Biological and Natural Sciences, Triângulo Mineiro Federal University, Uberaba, MG, Brazil; Ohio State University, UNITED STATES

## Abstract

Visceral leishmaniasis (VL) is a serious and fatal disease. Therapeutic drugs are toxic and non-sterilizing. The etiological agents *Leishmania infantum* and *Leishmania donovani* cause active and asymptomatic diseases. Effective drugs to treat VL exist but unfortunately, post-treatment relapses are common. Little is known why drugs are non-sterilizing or how these intracellular pathogens can escape treatment. Here, using a murine model of VL we found that CD271+/Sca1+ bone marrow mesenchymal stem cells (BM-MSCs) are readily infected *in vitro* and *in vivo* by *L*. *infantum*. Because BM-MSCs express potent drug efflux pumps, e.g., ABCG2 it is possible that this unique intracellular infectious niche could allow *L*. *infantum* to escape anti-parasite drugs.

## Introduction

Visceral leishmaniasis (VL) is caused by *Leishmania donovani-*complex organisms and is endemic in the tropical and subtropical areas of the World. VL presents high lethality, especially in untreated individuals, in malnourished children, and HIV-infected patients [1].

Once inside host cells, the parasites change from the promastigote to amastigote form, proliferate, and after lysis of the host cells again infect other target cells [2]. *Leishmania* parasites reside primarily inside Mononuclear Phagocytic System (MFS) cells [3]. However, other cells have also been reported to be susceptible to *Leishmania* infection such as fibroblasts [4], amniotic epithelial cells [5], human epithelial cells [5], hepatocytes [6] and adipose tissue-derived mesenchymal stem cells [7].

Mesenchymal stem cells (MSC) can be derived from a variety of different sources and are characterized as a population of cells with high proliferative capacity, that adhere to plastic, have specific surface antigen expression, and multipotent differentiation potential [8]. MSCs demonstrate an immunomodulatory role both *in vitro* and *in vivo* [9,10], and have been extensively studied due to their potential effectiveness for cellular therapy. Up until now, the CD271 marker has been proposed as one of the most specific marker for the purification and expansion of multipotent MSC from BM [10,11]. The CD271-MSCs grown without growth factors showed persistent CD271 expression, colony-forming unit fibroblast activity, high proliferative capacity and a greater capacity to give rise to adipocyte, as well as osteoblastic and chondroblast differentiation [10,11].

Recently, we have identified the CD271^+^ bone marrow mesenchymal stem cell (BM-MSC) population as a unique niche that harbors the intra-cellular pathogen *Mycobacterium tuberculosis* (*Mtb*) in humans with latent tuberculosis infection (LTBI) and in a mouse model of LTBI [12,13]. Indeed, these findings are consistent with many of the biological properties of BM-MSCs that point them as a unique niche that can provide protection to intracellular pathogens from the host response and from therapeutic drugs. First, these cells are present in addition to bone marrow in the lung and lymph node granulomas of infected mice and humans [14]. Second, stem cells possess the capacity for self-renewal [15,16]. Third, they express drug efflux pumps such as ABCG2 that could contribute to drug evasion by *Mtb* [17]. Fourth, they have low production of reactive oxygen species (ROS) which can favor the viability of non-replicating organisms [18]. Fifth, although stem cells have the capacity of self-renewing they are relatively quiescent [19] and reside in the immune privileged niche of the bone marrow [20,21]. Sixth, mesenchymal stem cells do not normally express MHC Class II on their cell surface and their MHC Class I molecules are functionally inactive, i.e., these molecules do not trigger effector functions of cytotoxic T lymphocytes [22].

Although the concept of latent infection with the causative agents of VL has been convincingly demonstrated [23] the host cell and parasite factors that contribute to this phase of the infectious process are not known. A fundamental question is the identification of the cell population that is used by these intracellular pathogens to allow them to escape the host immune response as well as to protect them from drugs. The present study suggests that BM-MSC may represent such a niche.

## Material and Methods

### Ethics statement

Male C57BL/6 mice, 5–8 weeks of age, were obtained from Charles River Laboratories and were maintained under pathogen-free conditions at The Forsyth Institute animal facility. The Forsyth Institutional Animal Care and Use Committee (IACUC) approved all procedures involving animals (Protocol #14–024 024–9/25/2014). All the experiments were performed in accordance with the approved guidelines of Forsyth’s IACUC and Institutional Biosafety Committee. Mice were housed in groups of 4 mice/cage. Rodent diet and reverse osmosis water were available ad libitum. Enrichment items were provided including mouse igloo and nesting material composed of shredded papers. The mice were checked daily by animal facility personnel and twice a week by the researchers. During the experiment we did not observe any animal deaths or animals exhibiting severe illness. We have a protocol in place for the use of humane endpoints but mice who are infected with Leishmania infantum do not typically become severely ill, therefore we did euthanized any of the mice prior to the experimental endpoint. At the end of the experiment euthanasia was performed using CO2. Following apparent clinical death, euthanasia was ensured by cervical dislocation.

### Parasites

*L*. *infantum chagasi* (MHOM/BR/00/1669), originally isolated from a Brazilian patient with visceral leishmaniasis was kindly supplied by Dr. Mary E. Wilson (University of Iowa, Iowa City, IA). Parasites were grown in hemoflagellate minimal essential medium (HOMEM) supplemented with 10% FBS, 100 U/ml penicillin-streptomycin. Cultures were monitored to ascertain that parasites had reached the stationary phase (7–10 days) before they were used in co-culture experiments with BM-MSC, as well as to infect mice.

### Generation of anti-L. infantum antibody

A rabbit anti-soluble lysate antigenic protein of *L*. *infantum* antiserum was prepared as we have previously described [24]. Briefly, 100μg of leishmanial proteins suspended in 1 ml of PBS were emulsified with 1 ml of incomplete Freünd’s adjuvant (IFA). The emulsion was injected in multiple sub-cutaneous (s.c.) sites into two female New Zealand rabbits. The rabbits were given two s.c. boosters (100μg of the antigen in IFA) six weeks apart followed by one intra-venous injection of 100μg of antigen. One week after the final boost, the rabbits were sacrificed by exsanguinations and sera were collected and stored at -20°C. Purified IgG from both the pre-immune and immune rabbit sera was prepared using *Staphylococcus aureus* protein A columns (MontageTM Antibody Purification, Millipore Corporation, Bedford, MA) as instructed by the manufacturer. Antibody activity of the purified IgG was confirmed by Flow Cytometry analysis (S1 Fig).

### Isolation of BM-MSCs and cultures

Bone marrow mononuclear cells (BM-MNCs) were isolated from mouse compact bones as recommended by EasySep^™^ Mouse Mesenchymal Stem/Progenitor Cell Enrichment Kit (kit #19771, STEMCELL Technologies, Vancouver, BC, Canada), which is designed to isolate mesenchymal stem/progenitor cells from mouse by negative immunomagnetic selection. Unwanted cells are targeted for removal with biotinylated antibodies that are directed against non-mesenchymal stem/progenitor cells markers CD45 and TER119. BM-MNCs were co-cultured with *L*. *infantum* promastigotes in RPMI supplemented with 10% FBS, 100 U/ml penicillin-streptomycin for 96 hours (MOI 5/1). Cultures were performed in 8-well cell chamber slides (Millicell^®^ EZslide 8 well glass- Merck Millipore). *L*. *infantum* promastigotes in stationary phase were washed two times with RPMI by centrifugation at 3000 rpm for 10 minutes and added to the 8-wells chamber slides containing the mammalian cells. The co-cultures were performed after one hour of cells isolation and then incubated in 5% CO_2_ at 37°C in a humidified incubator. After 96 hours, the wells were washed with PBS to remove the unattached parasites. Cells were fixed with 4% formaldehyde for 10 min, labeled with anti-CD271 (Abcam, ab27007), anti-Sca1 (Biolegend cn/ 122511 clone E13-61.7), anti-CD11b (Abcam, ab64347, Alexa 568), rabbit anti-*L*. *infantum* IgG antibodies followed by goat-anti rabbit IgG (Abcam, Alexa 488) and with DAPI and were then used for confocal staining.

### Experimental infection *in vivo*

Male C57BL/6 mice, 5–8 weeks of age were inoculated by intraperitoneal (i.p.) with 10^7^ *L*. *infantum* (MHOM/BR/00/1669) promastigotes (stationary phase). Animals were sacrificed 30 and 60 days post-infection. Bone marrow and spleen cells were analyzed to detect the presence of intracellular parasites.

## Results

### *L*. *infantum* infects CD271^+^ BM-MSCs *in vitro*

To determine if *L*. *infantum* can infect BMSCs, these cells were purified from non-infected mouse bone marrow using magnetic sorting protocol from Stem Cell Technologies Negative Selection kit. The cells were then added to chamber slides, exposed to virulent *L*. *infantum* promastigote parasites and cultured in complete RPMI medium at 37° for 96 hours. Cells from one half of the cultures were removed from the chambers followed by washing and staining for Flow Cytometry analysis. The supernatants from the second half of the cultures were gently removed and cultures were *in situ* gently washed with medium, followed by fixation with formaldehyde and staining with labeled antibodies specific for CD271, Sca1, CD11b and *L*. *infantum* antibody and with DAPI. Internalization of the parasites was then analyzed by confocal microscopy.

**Fig 1:**
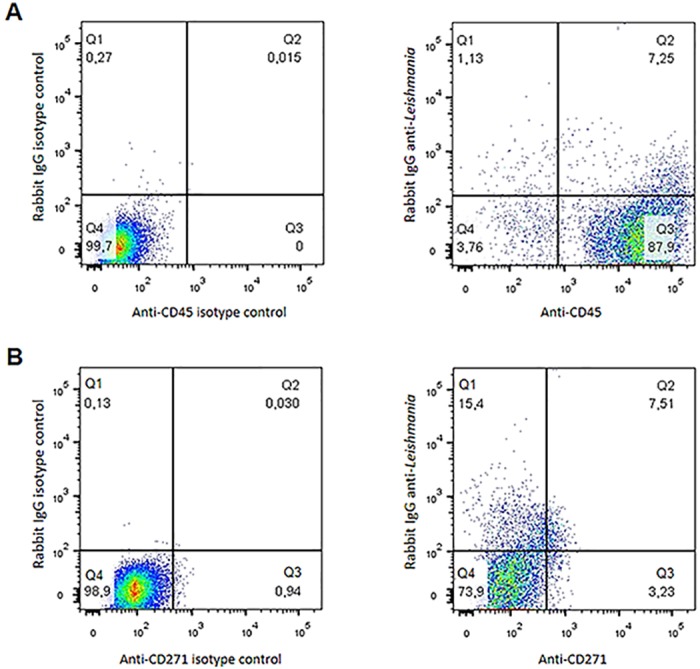
Flow cytometry analysis of mouse bone marrow mononuclear cells (BM-MNCs) co-cultured *in vitro* with *L*. *infantum*. Cells were co-cultured for 96h, stained in (A) with anti-CD45 (PE) and anti-*Leishmania* (FITC). In (B) cells were stained with anti-CD271+ (ALEXA 568) and anti-*Leishmania* (FITC). No staining with anti-leishmania-FITC was seen in all preparations using BM-MNCs cells not exposed to the parasites but stained with either anti-CD45 or anti-CD271 (not shown)

**Fig 2:**
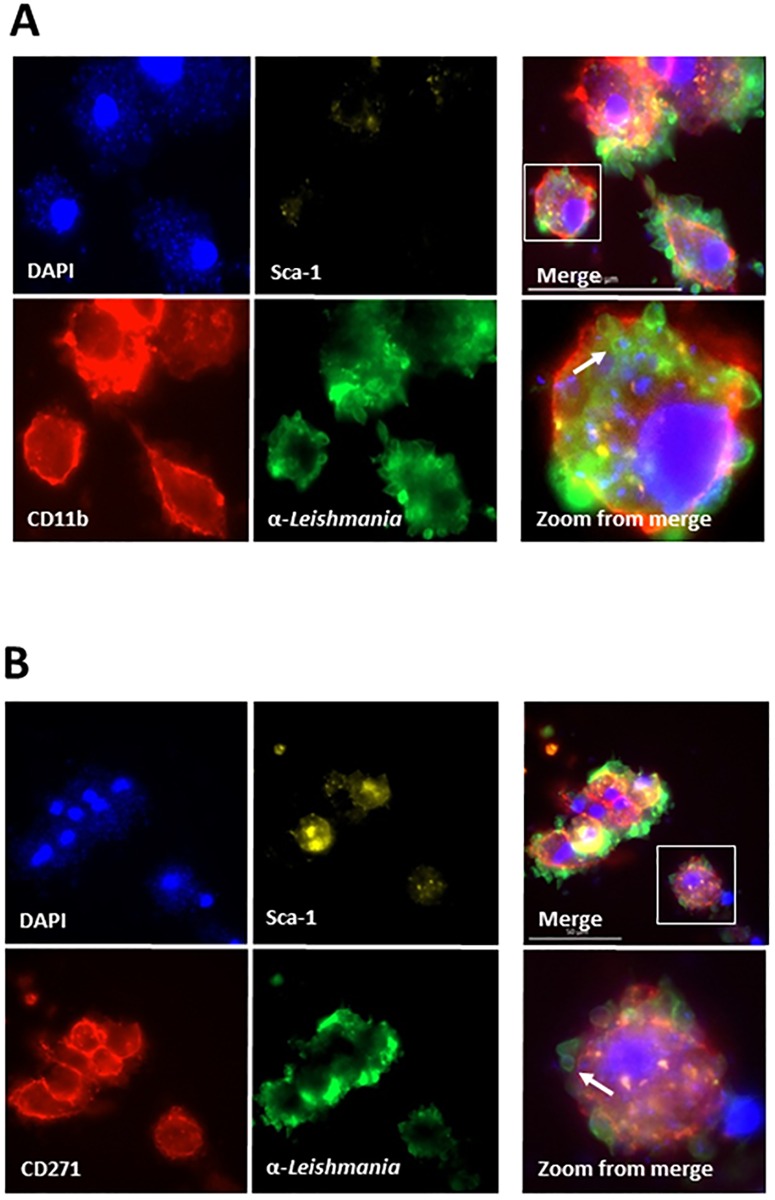
Confocal microscopy of mouse bone marrow mononuclear cells (BM-MNCs) co-cultured *in vitro* with *L*. *infantum*. Cells were co-cultured for 96h, stained with anti-CD11b (ALEXA 568), anti-CD271+ (ALEXA 568), Sca1 (APC) and anti-*Leishmania* (FITC). (A), BM-MNCs cells infected with *L*. *infantum* stained with anti-CD11b, Sca1, anti-leishmania and DAPI. (B), BM-MNCs cells infected with *L*. *infantum* stained with anti-CD271, Sca1, anti-leishmania and DAPI. No staining with anti-leishmania-FITC was seen in all preparations using BM-MNCs cells not exposed to the parasites but stained with anti-CD11b, Sca1 and antiCD271 (not shown). Arrows in Zoom from merge show one of several round images that clearly suggest amastigote forms of the parasite.

The Flow Cytometry analysis was initially performed to verify if the leishmania parasites could be detected in association with BM-MSCs. Cultures were stained with labeled antibodies specific to either CD45 (leukocyte common antigen) or CD271 cell surface markers. In addition, the cells were incubated with a rabbit IgG anti-leishmania antibody followed by addition of labeled goat anti-rabbit IgG. Cells were then analyzed by Flow Cytometry. Results are shown in Fig 1 and indicate that the leishmania parasites were associated with both the CD45+ cells (as expected) as well as, and importantly, with the CD271+ cells. Approximately 7.25% of the CD45+ cells and 7,5% of the CD271+ cells were also stained with the anti-leishmania antibody. These results suggest that, like *M*. *tuberculosis*, *L*. *infantum* may also use BM-MSCs as an infectious niche. To have a morphological and better definition of interaction of the parasites with host cells we next used confocal microscopy. This analysis revealed, again as expected, that the CD11b+ cells (macrophages) were clearly infected (Fig 2A). But importantly, the CD45-, Sca1+, CD271+ cell population (BM-MSCs) was equally infected with amastigote-like forms of *L*. *infantum* (Fig 2B) thus suggesting that *L*. *infantum* can also infect BM-MSCs. Because the leishmania structures seen in the preparations stained with either anti-CD11b or anti-CD271 antibodies are amastigote forms of the parasites (not the promastigote forms used for infection), the results point to the intracellular localization of *L*. *infantum* and not simple adsorption to the surfaces of the host cells. Non-infected control cells staining with anti-CD11b or CD271 and Sca1 did not stain with anti-*Leishmania* antibody thus confirming the specificity of the confocal microscopy (not shown).

### *L*. *infantum* infects CD271^+^ BM-MSCs *in vivo*

**Fig 3:**
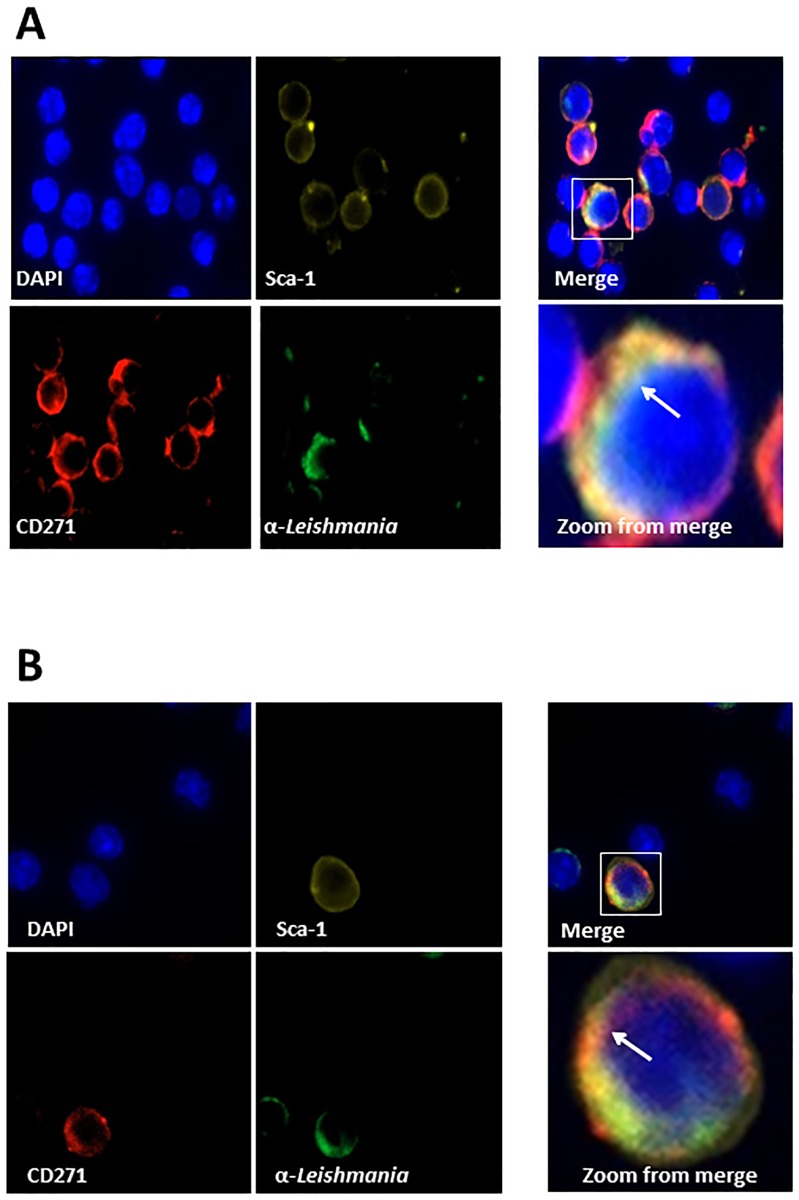
Confocal microscopy of mouse bone marrow mononuclear cells (BM-MNCs) from bone marrow of mice infected with *L*. *infantum*. Mice were infected with promastigote forms of *L*. *infantum* and sacrificed 30 days (A) or 60 days (B) later. Bone marrow cells were obtained and enriched for CD45- CD271+ cells. Cells were incubated in 8-well chamber slides to adhere followed by staining with DAPI, anti-CD271, anti-Sca1 and rabbit anti-*L*. *infantum* IgG. Note in Merge clear images that point to the presence of amastigote forms stained with anti-leishmania FITC antibody associated with CD271+, Sca1+ cells. Arrows in Zoom from merge shows one of several round images that clearly suggest amastigote forms of the parasite. No staining with anti-leishmania-FITC was seen in all preparations using cells from non-infected mice (not shown)

**Fig 4:**
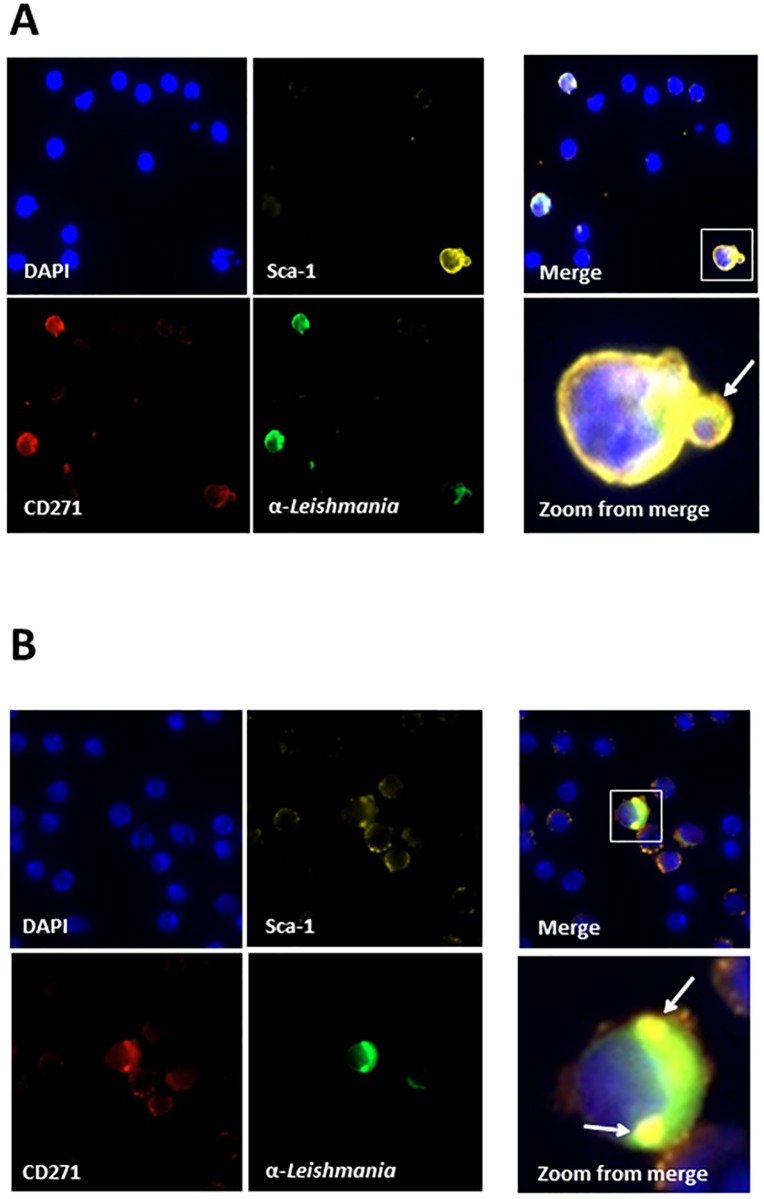
Confocal microscopy of mouse spleen mononuclear cells from mice infected with *L*. *infantum*. Mice were infected with promastigote forms of *L*. *infantum* and sacrificed 30 days (A) or 45 days (B) later. Spleen mononuclear cells were obtained and enriched for CD45- CD71+ cells. Cells were incubated in 8-well chamber slides to adhere followed by staining with DAPI, anti-CD271, anti-Sca1 and rabbit anti-*L*. *infantum* IgG. Note in Merge clear images that point to the presence of amastigote forms stained with anti-leishmania FITC antibody associated with CD271+, Sca1+ cells. Arrows in Zoom from merge shows one of several round images that clearly suggest amastigote forms of the parasite. No staining with anti-leishmania-FITC was seen in all preparations using cells from non-infected mice (not shown).

The presence of *Leishmania* amastigote organisms in spleen, liver, and bone marrow macrophages of VL patients is a well-known condition of this pathology in humans. Therefore to begin to evaluate the possibility that MSCs present in both bone marrow and spleen are also a target of the infectious process *in vivo*, C57BL/6 mice were inoculated with 10^7^ promastigote forms of *L*. *infantum* (stationary phase). Animals were then, euthanized at different time points after challenge (30 and 60 days post-infection). To confirm the infection, animals were sacrificed and spleen inprints were stained with Giemsa followed by conventional microscopic examination and detection of typical leishmanial amastigotes. Moreover, the degree of intracellular infection of the spleen cells was evaluated by Flow Cytometry using both anti-CD45 and anti-leishmania antibodies. S2 Fig illustrates the results and confirm that approximately 4% of the CD45+ cells were also stained with anti-leishmania antibody, thus confirming the *in vivo* infection. The presence of parasite inside CD271^+^ Sca1^+^ cells was investigated using confocal microscopy in both spleen and BM cells. Similar to the confocal experiments performed with *in vitro* cultures, before staining with the specific antibodies the CD271+CD45- cells were enriched using the Stem Cell Technologies Negative Selection kit (#19771). Figs 3 and 4 illustrate the results and clearly show that CD271^+^ Sca1^+^cells from both BM and spleen harvested 30 and 60 days post challenge have internalized amastigote-like forms of the parasite.

To verify that viable *L*. *infantum* could be retrieved from infected BM-MSCs, CD271+CD45- cells were obtained from the bone marrow of infected mice at 30 and 60 days post challenge. Purified cells were then inoculated at 26°C in complete HOMEM *Leishmania* culture medium for seven days, after which time abundant motile promastigote forms of the parasite were readily detected by direct microscopic examination of the cultures from both time points (S1 Table). Although the Easysep kit used for the purification of the BM-MSCs does not yield highly purified BM-MSCs these results suggest that viability of *L*. *infantum* is maintained *in vivo* in infected CD271+CD45- cells. However, at this point, we can not categorically affirm that viable *L*. *infantum* were recovered from BM-MSCs and not from possibly contaminating CD11b cells.

Nonetheless, together these findings indicate that in the mouse model of VL *L*. *infantum* infects BM-MSCs and suggest that, like *Mtb* [12,13], the parasite could utilize BM-MSCs as a possible mechanism of escapefrom drugs.

## Discussion

VL remains endemic in many tropical and subtropical areas of the World with many challenges facing the successful control of this disease. One of the major challenges to control this disease is the lack of basic knowledge about the mechanisms by which *L*. *donovani* complex organisms persist in a “latent-like” stage within the host during subclinical conditions.

Although macrophages are known to be the primary host cell for *Leishmania* parasites [3], several other cell types have been shown to be able to harbor *Leishmania in vitro* or *in vivo*, such as neutrophils, eosinophils, dendritic cells, fibroblasts, amniotic epithelial cells, human epithelial cells, hepatocytes and adipose tissue derived mesenchymal stem cells [4–7]. However, the viability of *Leishmania* in most of these intracellular niches is poor and because most of these cells are short lived no evidence exists indicating that they can maintain *in vivo* live parasites for long periods of time. Hence, these cell types, including macrophages and dentdritic cells are unlikely candidates that may serve as a reservoir for live parasites *in vivo* during latent infection.

Recently, we have shown that mesenchymal stem cells (MSCs) may provide to *M*. *tuberculosis* a protective niche from the host immune response and from drugs during latent infection and active disease [12,13]. We proposed that this unique host/pathogen escape mechanism could be broadly applied to other intracellular pathogens. MSCs have been extensively studied due to their multipotent and immunomodulatory characteristics. These cells possess specific markers as Stro-1, SSEA-4, CD271, CD146, and Sca1, but the expression of these markers is different in cells obtained from various sources [25]. Although CD271 marker has failed to detect cord blood mesenchymal stem cells, CD271 have been considered the most genuine and useful molecule to isolate and detect MSCs from BM [11,26,27].

BM-MSCs may provide an ideal protective niche for *Leishmania* because these cells have several properties that are critical for the pathogen’s long-term persistence and survival. First, BM-MSCs do not normally express MHC Class II on their cell surface; in addition their MHC Class I molecules, similar to a number of other cell populations do not trigger effector functions of cytotoxic T lymphocytes [28]; and second, BM-MSCs reside in the immune privileged or immune evasive niche in the bone marrow [20,29]. These properties confer to these cells protection from immune attack. In addition, BM-MSCs have the capacity for self-renewal [16,30], are relatively quiescent [19] and have low reactive oxygen species [31] properties that might benefit long term viability of the parasites. Finally, and importantly they express potent drug efflux pumps such as ABCG2 [32] that could contribute to drug evasion by *Leishmania* organisms.

Therefore, we set out to examine whether these cells could be a host for long term persistence of *L*. *infantum*. We confirmed that the MSC CD271+ Sca1 cells from mice BM and spleen can indeed harbor the parasites as indicated by both *in vitro* and *in vivo* experiments. A preliminary study suggested that adipose tissue derived mesenchymal stem cells (ADMSCs) could be infected *in vitro* by different species of *Leishmania* [7]. The present results expand these observations and clearly show that *L*. *infantum* can infect BM-MSCs both *in vitro* and *in vivo*. However, it remains to be investigated the relative proportion of infected mesenchymal cells present in the spleen and bone marrow compared to the conventional host cells like macrophages and dendritic cells. Based on the overall low numbers of mesenchymal stem cells normally present in these organs it is likely that infection of these cells with *L*. *infantum* represents only a fraction of the whole process. However, this targeted infected cell population is sufficient for the establishment and maintenance of the latent infectious process as we have previously shown to occur in humans and mice infected with *M*. *tuberculosis* [12,13]. In other words, macrophages and dendritic cells are the major targets of the infection process that accompany the pathology of active VL and BM-MSCs are the targeted infected cells present in asymptomatic or latent VL.

Epidemiological studies indicate that the high VL recurrence following drug treatment remains a major challenge [33,34]. Therefore, prevention and management of the re-activation process could reduce the incidence of recurrent VL. However, the host cells and mechanisms of reactivation are not clearly known, which compromises our ability to control VL.

We have demonstrated for the first time that *L*. *infantum* can infect CD45-, CD271+, Sca1+ cells both *in vitro* and *in vivo*. These results support our proposed hypothesis that *L*. *infantum* parasites could hijack themselves inside of MSCs in BM and spleen, possibly facilitating an infectious process that could lead to asymptomatic disease. MSC cells might form a less hostile environment for *L*. *infantum* than macrophages and thereby allow for the persistence and resistance of the parasites. Moreover, because MSCs express potent drug efflux pumps such as ABCG2 it is possible that these cells could provide for the parasite an important mechanism of drug evasion, which is a common condition observed in VL treated patients.

In conclusion, we have shown that BM-MSCs can harbour *Leishmania* parasites following *in vitro* and *in vivo* infection. However, whether BM-MSCs protect this pathogen to evade drugs or the immune response, as they do to *M*. *tuberculosis*, remains a speculation. Experiments are in progress to substantiate this possibility.

## Supporting Information

S1 Fig Anti-*L*. *infantum* activity of purified rabbit IgG specific antibody measured by Flow Cytometry analysis. Promastigote forms of the parasite were incubated with 0.2 ug/ml of purified IgG followed by a second incubation with fluorescein labelled goat anti-rabbit IgG. Antibody activity was subsequently analyzed by flow cytometry.
(TIF)

S2 Fig Evaluation of infection in mice inoculated with *L*. *infantum*. Animals were inoculated i.v. with 107 promastigotes of L. infantum and sacrificed 30 days later. Spleen cells were obtained followed by staining with anti-CD45 (PE labeled) and anti-Leishmania (FITC labeled) antibodies and subsequently analyzed by Flow Cytometry.
(TIF)

S1 Table Retrieval of viable L. infantum from CD271+CD45- cells purified from bone marrow of infected mice. (DOCX)
