# Supplementary figures and images for: CD271+ Mesenchymal Stem Cells as a Possible Infectious Niche for Leishmania infantum
Source: PLoS One. 2016 Sep 13;11(9):e0162927. doi: 10.1371/journal.pone.0162927 (PMC5021359; doi:10.1371/journal.pone.0162927)

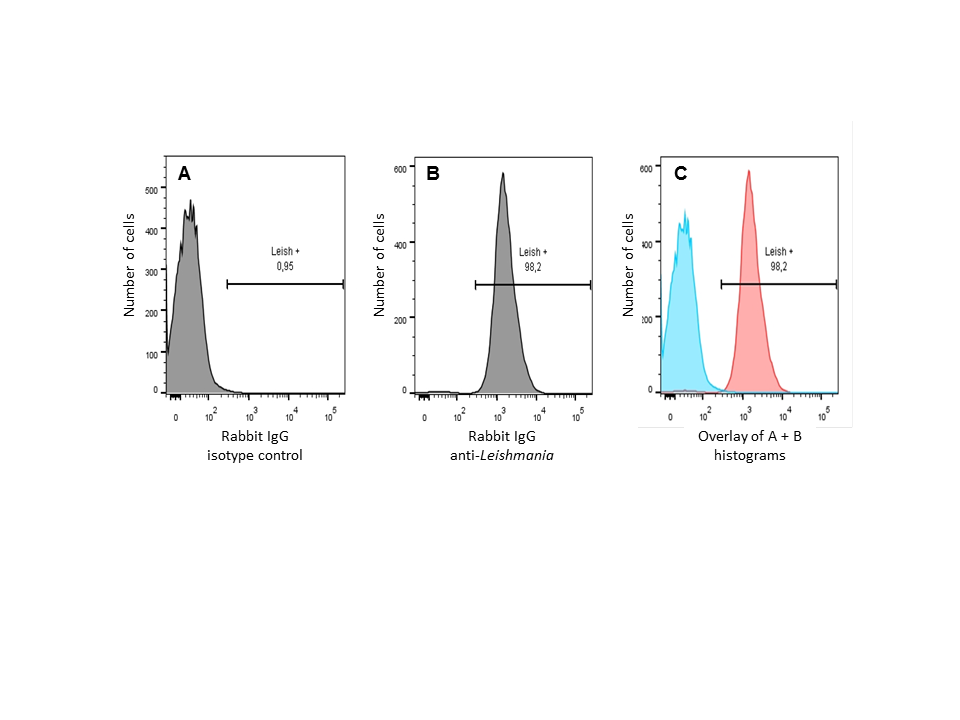

Supplement: S1 Fig — Promastigote forms of the parasite were incubated with 0.2 ug/ml of purified IgG followed by a second incubation with fluorescein labelled goat anti-rabbit IgG. Antibody activity was subsequently analyzed by flow cytometry. (TIF) [file pone.0162927.s001.TIF]

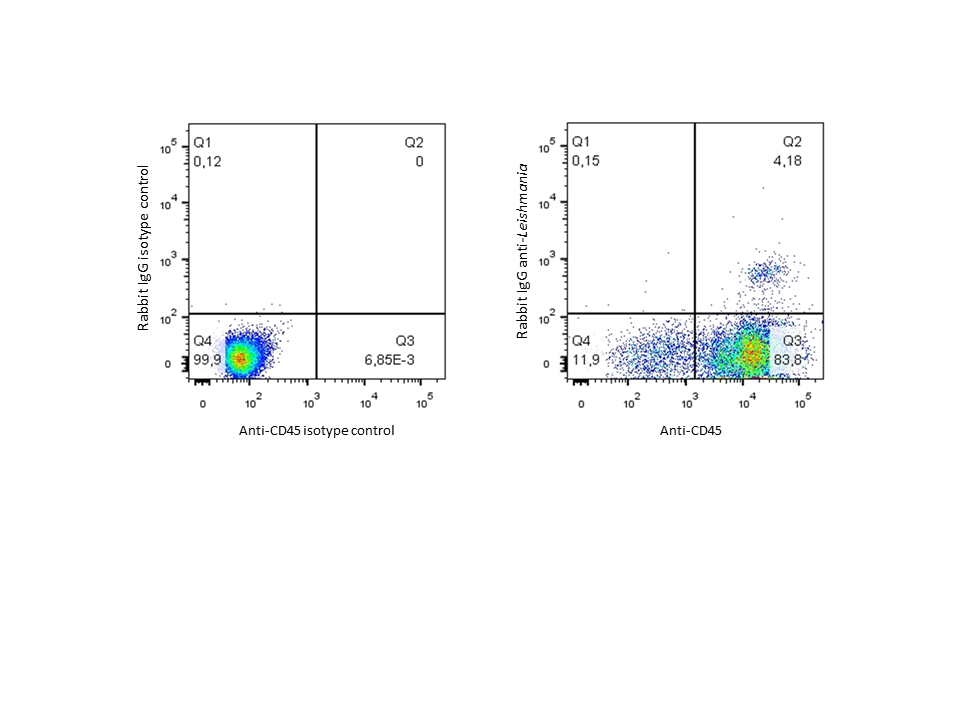

Supplement: S2 Fig — Animals were inoculated i.v. with 107 promastigotes of L. infantum and sacrificed 30 days later. Spleen cells were obtained followed by staining with anti-CD45 (PE labeled) and anti-Leishmania (FITC labeled) antibodies and subsequently analyzed by Flow Cytometry. (TIF) [file pone.0162927.s002.TIF]
