# Supplementary material for: CD271+ Mesenchymal Stem Cells as a Possible Infectious Niche for Leishmania infantum
Source: PLoS One. 2016 Sep 13;11(9):e0162927. doi: 10.1371/journal.pone.0162927 (PMC5021359; doi:10.1371/journal.pone.0162927)
Supplement: S1 Table — (DOCX) [file pone.0162927.s003.docx]

| **Table 1. Retrieval of viable *L. infantum* from CD271+CD45- cells purified from bone marrow of infected mice***. | | |  |
| --- | --- | --- | --- |
|  | Time after infection (days) | |  |
|  | 30 | 60 | |
| Parasite growth | +++ | +++ | |
| (*) Purified CD271+CD45- cells were inoculated in complete HOMEM Leishmania culture medium at 26^o^C for seven days, after which time motile promastigote parasite growth (+++) was detected by direct microscopic examination of the cultures. | | |  |
